# A Comprehensive Analysis of Interaction and Localization of Arabidopsis SKP1-LIKE (ASK) and F-Box (FBX) Proteins

**DOI:** 10.1371/journal.pone.0050009

**Published:** 2012-11-15

**Authors:** Hirofumi Kuroda, Yuki Yanagawa, Naoki Takahashi, Yoko Horii, Minami Matsui

**Affiliations:** 1 Plant Functional Genomics Research Group, Plant Science Center, RIKEN Yokohama Institute, Yokohama, Kanagawa, Japan; 2 Synthetic Genomics Research Team, Biomass Engineering Program, RIKEN Yokohama Institute, Yokohama, Kanagawa, Japan; Iwate University, Japan

## Abstract

F-Box (FBX) proteins are encoded by a multigene family present in major lineages of eukaryotes. A number of FBX proteins are shown to be subunits of SCF complex, a type of E3 ligases composed of SKP1, CULLIN, FBX and RBX1 proteins. The Arabidopsis SKP-LIKE (ASK) proteins are also members of a family and some of them interact with FBX proteins directly. To clarify how FBX and ASK proteins combine, we carried out a large-scale interaction analysis between FBX and ASK proteins using yeast *two-hybrid* assay (Y2H) in *Arabidopsis thaliana*. FBX proteins randomly chosen from those proteins that interacted with more than one ASK protein were further analyzed for their subcellular localization and *in vivo* interaction with ASK proteins. Furthermore, the expression profiles of *FBX* and *ASK* genes were compared. This work reveals that FBX proteins had a preference for interacting with ASK proteins depending on the domains they contain such as the FBX-associated (FBA) domain, the Kelch domain and leucine rich repeat (LRR). In addition, it was found that a single FBX protein could form multiple SCF complexes by interacting with several ASK proteins in many cases. Furthermore, it was suggested that the variation of SCF complexes were especially abundant in tissues related to male gametophyte and seed development. More than half of the FBX proteins studied did not interact with any of the ASK proteins, implying the necessity for certain regulations for their interaction *in vivo* and/or distinct roles from subunits of the SCF complex.

## Introduction

Protein regulation is known to be an important system to allow adaptation to various abiotic and biotic stresses such as heat, drought and pathogens as well as for carrying out normal functions for survival. Ubiquitin (Ub)-mediated regulation is one of the key mechanisms for degradation and protein signaling in eukaryotes. In plants, various proteins are regulated by the Ub-mediated system in response to different environmental stresses and developmental signals such as the cell cycle and flowering [1], [2], [3], [4], [5], [6].

Ub is a small protein composed of 76 amino acids and is highly conserved in eukaryotes. The Ub molecule is attached to its target protein through the sequential actions of three enzymes, Ub-activating enzyme (E1), Ub-conjugating enzyme (E2) and Ub ligase (E3). E3 ligase has an especially diverse gene family in plants, comprising more than 1,400 genes as predicted in Arabidopsis [7], [8]. Of the several types of E3 ligases, the SCF complex, composed of CULLIN, SKP1, RBX1 and FBX proteins, is the most variable. Since FBX proteins carrying the FBX domain, located mostly in the N-terminal region, function as receptors for recruitment of particular substrates for ubiquitination, FBX protein is thought to be a key factor conferring variable specificity against the substrate. Indeed, multiple *FBX* genes have been isolated from yeast (20 genes in *Saccharomyces cerevisiae*, 17 genes in *Schizosaccharomyces pombe*), fruit fly (27 genes in *Drosophila melanogaster*), and human (69 genes) [9]. Numerous *FBX* genes have been identified particularly in plants such as Arabidopsis (897 genes), rice (971 genes) and popular (425 genes) [10], because functional diversity within a gene family is thought to develop a prompt response to environmental changes in addition to the various signals involved in plant development [11], [12].

Arabidopsis has 21 *Skp1* homologs, *ASK*s, in its genome. Of these, ASK1 has been well characterized and its interaction with several kinds of FBX proteins such as TIR1 and COI1 has been reported [13], [14], [15], [16]. The *ask1* mutation caused male sterility and ASK1 is essential for early nuclear reorganization in male meiocytes [17], [18]. Moreover, a proteomic approach revealed that the amounts of protein involved in photomorphogenesis, circadian oscillation, post-translation processes, stress responses and cell expansion or elongation were altered in the *ask1-1* mutant compared to wild type, implying multiple physiological roles of ASK1-mediated protein regulation [19]. The ASK2 protein, which has the most similar sequence to ASK1, is also known to function in male meiosis [20]. Moreover, the *ask1 ask2* double mutant showed a developmental retardation during embryogenesis and lethality at the seedling stage [21].

Previously, we and another group reported that the 19 *ASK* genes (*ASK1-19*) had different patterns for expression in almost all the tissues examined [15], [22]. In addition, we observed that 9 FBX proteins interacted with more than one ASK protein in yeast *two-hybrid* assays (Y2H) [15], [16]. In this study to further elucidate this specificity between FBX and ASK proteins, we performed a large-scale analysis of the interaction of 341 FBX proteins with 19 ASK proteins by Y2H. In addition, we compared the gene expression patterns between *FBXs* and *ASKs* in several tissues using microarray data from a gene expression search engine. Moreover, subcellular localization and *in vivo* interaction with ASK proteins were also examined on randomly chosen FBX proteins. These results enable us to predict the physiological functions of SCF complexes including FBX and ASK proteins in tissues and/or cell compartments. This study provides helpful information for further work into Ub-mediated protein regulation through SCF complexes in plants.

## Results

### Comprehensive interaction maps between FBX and ASK proteins of Arabidopsis

Recent progress in full-length cDNA, ESTs, and genome information has much facilitated analysis in plants. To generate an interaction map between FBX and ASK proteins of Arabidopsis, we cloned 341 cDNAs of Arabidopsis *FBXs* including previously cloned genes [15], [16] and classified them by the variable domains of their translated proteins, located mostly in the C-terminal region, according to Hua et al [10].

**Figure 1:**
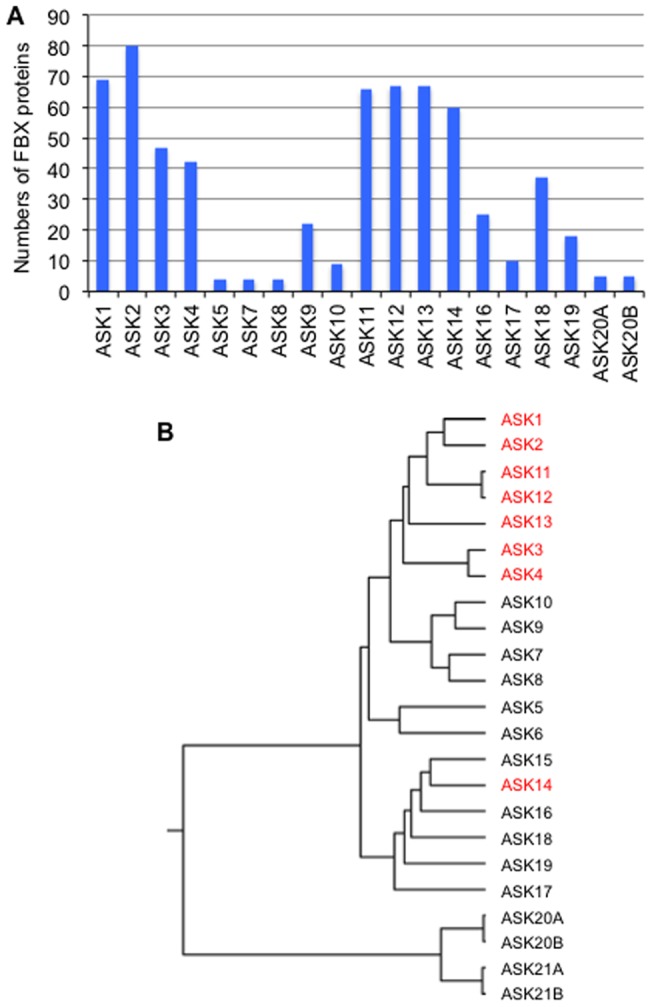
Y2H between FBX and ASK proteins. (A) Number of FBX proteins that interacted with each ASK protein. (B) Phylogeny of ASK proteins using the unweighted pair group method with arithmetic mean of Clustal W (http://www.genome.jp/tools/clustalw/). ASK proteins, which interacted with more than 40 FBX proteins, are showed in red.

Y2H was performed between 341 FBX and 19 ASK (1–5, 7–14, 16–19, 20A and 20B) proteins (Table S1 and Figure 1). Of these, 140 FBX proteins interacted with at least one ASK protein. Interestingly, specificity against FBX proteins was observed in ASK proteins; eight ASK proteins (1–4 and 11–14) interacted with more than 40 FBX proteins, while other ones interacted with far fewer (Figure 1A). These eight ASK proteins had great similarity in their amino acid sequences, with the exception of ASK14 (shown as red in Figure 1B). Unexpectedly, more than half the FBX proteins (201 proteins) did not interact with any ASK proteins (Table S1).

**Figure 2:**
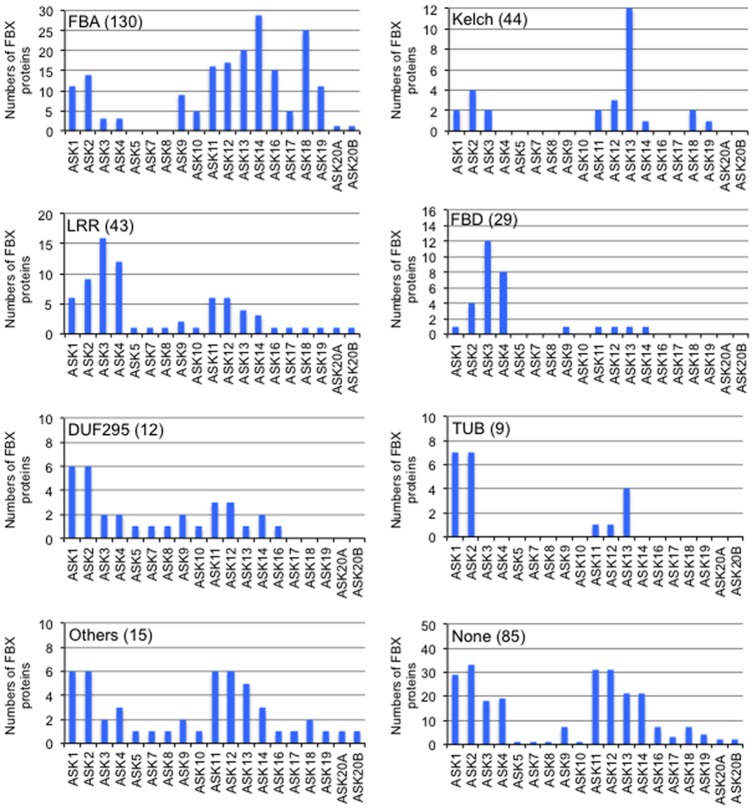
Relationship between variable domains of FBX proteins and ASK proteins by Y2H. Total numbers of FBX proteins carrying each domain (FBA, Kelch, LRR, FBD, DUF295, TUB and others) or none are indicated in parenthesis. Note that the number includes overlap of FBX proteins carrying multiple domains.

Since the distinct domains of FBX proteins are supposed to interact with various proteins, the types of FBX proteins as classified by the domains present may affect their selective interaction with ASK proteins. As shown in Figure 2 and Table S1, there were 256 FBX proteins carrying various domains; FBA (total number 130), Kelch (44), LRR (43), FBD (29), DUF295 (12), Tubby (TUB) (9) and/or other domains (15), in addition to FBX and/or FBX-like domains. As expected, each type of FBX protein had a preference for interacting with ASK proteins (Figure 2). FBX proteins with a FBA domain, which were the largest population, preferred ASKs 1–2, 9, 11–16 and 18–19. FBX proteins carrying LRR and FBD domains showed preference for ASKs 3–4, while ones carrying DUF295 and TUB domains preferred ASKs 1-2. FBX proteins with a Kelch domain had high specificity to ASK13. Eighty-five FBX proteins had no additional domain, and 45 FBX proteins of them could interact with more than one ASK protein.

### *In vivo* interaction between FBX and ASK proteins

**Figure 3:**
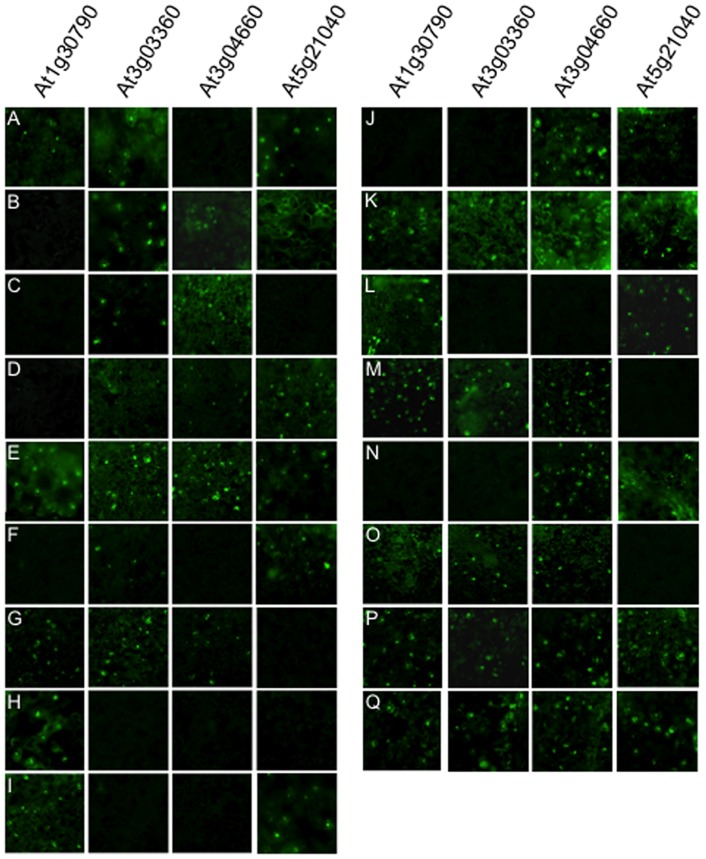
Interaction of FBX and ASK proteins *in vivo*. BiFC analyses were performed using combinations of four FBX (At1g30890, At3g03360, At3g04660 and At5g21040) (At1g30790, At3g03360, At3g04660 and At5g21040) and ASK proteins. (A) ASK1; (B) ASK2; (C) ASK3; (D) ASK4; (E) ASK5; (F) ASK7; (G) ASK8; (H) ASK10; (I) ASK11; (J) ASK13; (K) ASK14; (L) ASK16; (M) ASK17; (N) ASK18; (O) ASK19; (P) ASK20A; (Q) ASK20B.

*In vitro* interaction between FBX and ASK proteins was shown by Y2H. To examine that these results reflected the *in vivo* interactions, we performed bimolecular fluorescent complementation (BiFC) analysis using a transient assay system in *Nicotiana tabacum* (Figure 3). For this purpose, four FBX proteins, At1g30790, At3g03360, At3g04660, At5g21040, were randomly chosen (Tables 1 and S1). Interestingly, this analysis revealed that all four FBX proteins interacted with more ASK proteins than was shown by Y2H. At1g30790, which interacted with ASKs 14 and 16 by Y2H, also showed interaction with ASKs 1, 5, 8, 10–11, 17, 19, 20A and 20B in addition to ASKs 14 and 16 by BiFC analysis. At3g04660, shown to interact with ASKs 13 and 14 by Y2H, also interacted with ASKs 2–5, 8, 17–19, 20A and 20B in BiFC analysis. At5g21040, which interacted with ASKs 1–2 and 11–14 by Y2H, showed interaction with ASKs 4–5, 7, 16, 18, 20A and 20B in addition to ASKs 1–2, 11 and 13–14 by BiFC analysis, although no result was obtained with ASK 12. BiFC analysis indicated that At3g03360 interacted with ASKs 1–5, 7–8, 14, 17, 19, 20A and 20B *in vivo*, although ASK 16 was also shown to be an interacting partner by Y2H.

**Table 1:** Comparison between in vitro and in vivo interaction of randomly chosen FBXs and ASKs.

AGI	Analysis	ASKs^a^																		
		1	2	3	4	5	7	8	9	10	11	12	13	14	16	17	18	19	20A	20B
At1g30790	Y2H													+	+					
	BiFC	+				+		+		+	+	nd		+	+	+		+	+	+
At3g03360	Y2H													+	+					
	BiFC	+	+	+	+	+	+	+				nd		+		+		+	+	+
At3g04660	Y2H												+	+						
	BiFC		+	+	+	+		+				nd	+	+		+	+	+	+	+
At5g21040	Y2H	+	+								+	+	+	+						
	BiFC	+	+		+	+	+				+	nd	+	+	+		+		+	+

a “+” indicates the interaction between experimented FBXs and ASKs. nd; no data.

### Comparison of the gene expression of *FBXs* and *ASKs* in various tissues

**Figure 4:**
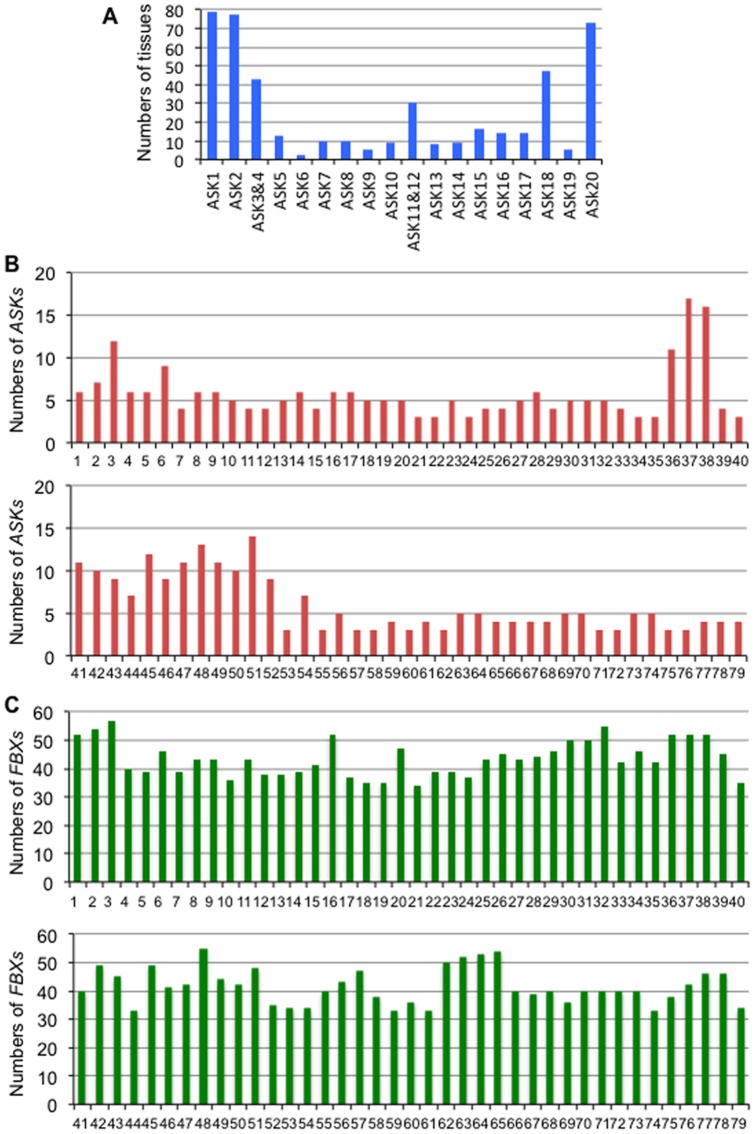
Comparison of gene expression of *ASK* and *FBX* in various tissues using microarray data of GENEVESTIGATOR. (A) Numbers of tissues where expression of *ASK* genes was observed were counted based on their expression profiles in Figure S1. (B) Numbers of *ASK* genes whose expression was observed in each tissue were counted based on their expression profiles in Figure S1. Numbers correspond to the tissue; callus (1), cell culture/primary cell (2), sperm cell (3), protoplast (4), guard cell protoplast (5), mesophyll cell protoplast (6), root protoplast (7), root cap protoplast (8), columella protoplast (9), lateral root cap protoplast (10), root epidermis and lateral root cap protoplast (11), root cortex protoplast (12), root endodermis and quiescent center protoplast (13), root stele protoplast (14), root phloem protoplast (15), root xylem protoplast (16), root cortex, endodermis and quiescent center protoplast (17), root epidermis protoplast (18), root epidermal atrichoblast protoplast (19), root culture (20), seedling (21), cotyledon (22), hypocotyl (23), radicle (24), imbibed seed (25), shoot apical meristem (26), inflorescence (27), flower (28), pistil (29), carpel (39), ovary (31), ovule (32), stigma (33), petal (34), sepal (35), stamen (36), anther (37), pollen (38), abscission zone (39), pedical (40), silique (41), replum (42), seed (43), embryo (44), endosperm (45), micropylar endosperm (46), peripheral endosperm (47), chalazal endosperm (48), testa (49), general seed coat (50), chalazal seed coat (51), suspensor (52), stem (53), developing meristemoid zone (54), node (55), shoot apex (56), cauline leaf (57), rosette (58), juvenile leaf (59), adult leaf (60), petiole (61), senescent leaf (62), hypocotyl (63), xylem (64), cork (65), leaf primordial (66), stem (67), axillary bud (68), axillary shoot (69), shoot apex (70), roots (71), primary root (72), root tip (73), meristematic zone (74), elongation zone (75), root hair zone (76), stele (77), pericycle (78) or lateral root (79). (C) Numbers of *FBX* genes whose expression was counted in each tissue based on their expression profiles in Figure S2. Numbers correspond to each tissue described in (B).

**Figure 5:**
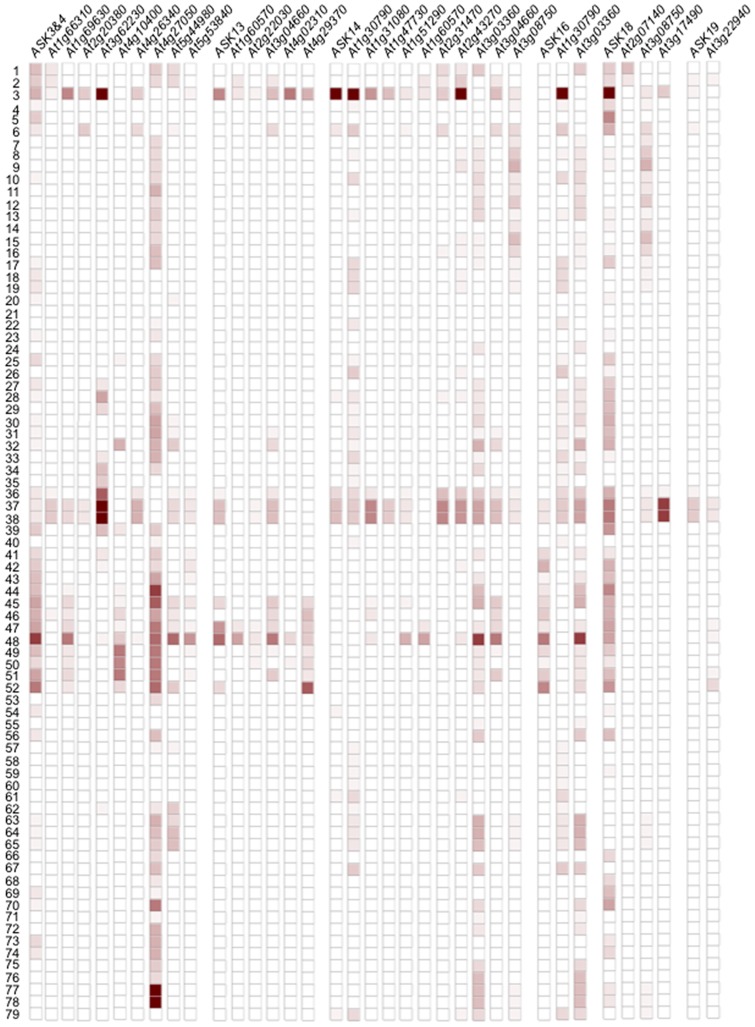
Comparison of expression patterns of *FBX* and *ASK* genes in various tissues using microarray data of GENEVESTIGATOR. Columns showing co-expression patterns between *FBX* and *ASK* genes are indicated by comparing the microarray data from Figures S1 and S2. Numbers correspond to the tissue; callus (1), cell culture/primary cell (2), sperm cell (3), protoplast (4), guard cell protoplast (5), mesophyll cell protoplast (6), root protoplast (7), root cap protoplast (8), columella protoplast (9), lateral root cap protoplast (10), root epidermis and lateral root cap protoplast (11), root cortex protoplast (12), root endodermis and quiescent center protoplast (13), root stele protoplast (14), root phloem protoplast (15), root xylem protoplast (16), root cortex, endodermis and quiescent center protoplast (17), root epidermis protoplast (18), root epidermal atrichoblast protoplast (19), root culture (20), seedling (21), cotyledon (22), hypocotyl (23), radicle (24), imbibed seed (25), shoot apical meristem (26), inflorescence (27), flower (28), pistil (29), carpel (39), ovary (31), ovule (32), stigma (33), petal (34), sepal (35), stamen (36), anther (37), pollen (38), abscission zone (39), pedical (40), silique (41), replum (42), seed (43), embryo (44), endosperm (45), micropylar endosperm (46), peripheral endosperm (47), chalazal endosperm (48), testa (49), general seed coat (50), chalazal seed coat (51), suspensor (52), stem (53), developing meristemoid zone (54), node (55), shoot apex (56), cauline leaf (57), rosette (58), juvenile leaf (59), adult leaf (60), petiole (61), senescent leaf (62), hypocotyl (63), xylem (64), cork (65), leaf primordial (66), stem (67), axillary bud (68), axillary shoot (69), shoot apex (70), root (71), primary root (72), root tip (73), meristematic zone (74), elongation zone (75), root hair zone (76), stele (77), pericycle (78) or lateral root (79). Percentage expression potential is shown from 0 (white) to 100 (dark brown) % according to the six-grade system.

To understand the physiological role of the interaction between FBX and ASK proteins, we examined expression patterns in various tissues of *ASK* and *FBX* genes whose translated products interacted with more than one ASK protein using the gene expression search engine GENEVESTIGATOR (Figures 4–5, S1–3). As shown in Figures 4A and S1, *ASK* genes were expressed in a variety of tissues, and each expression pattern was distinct to 2 types; one is expressed in a large number of tissues (*ASKs* 1–2, 3–4, 11–12, 18 and 20), and the other is expressed with much higher specificity in some tissues (*ASKs* 5–10, 13–17 and 19). Moreover, the former can be classified into 2 types; one is expressed in all tissues (*ASKs* 1–2 and 20), and the other is expressed in active tissues where cell division is occurring, including mitosis and meiosis in particular (*ASKs* 3–4, 11–12 and 18). The latter expression pattern can be further classified into 3 types; one is expressed preferentially in male tissues such as pollen and sperm cells (*ASKs* 6, 14 and 19), the second is expressed preferentially in tissues related to seed development such as siliques and endosperm (*ASKs* 7–10 and 16–17), and the third is expressed in both male tissues and tissues related to seed development (*ASKs* 5, 13 and 15). Considering the expression of *ASKs* on tissue side, more than three *ASK* genes were expressed in every tissue, and in particular large numbers of *ASK* genes were expressed in male tissues and tissues related to seed development such as siliques, endosperm and testa (Figure 4B).

The expression patterns of *FBX* genes whose translated products interacted with more than one ASK protein were also classified as being in a large number of tissues or specific tissues (Figure S2), although some had a lack of microarray data in a variety of tissues (Figure S3). However, compared to the expression levels of the *ASK* genes, there was not a large difference in the number of *FBX* genes expressed in each of the tissues observed (Figure 4C). Taking the tissue expression data together with the results of the Y2H, it is possible to predict the physiological functions of the various SCF complexes that include FBX and ASK proteins. The expression patterns of the *FBX* and *ASK* genes and the interaction profiles of their proteins by Y2H indicate that 25 *FBX* genes had correlative expression patterns to *ASKs 3, 4, 13, 14, 16, 18* and/or *19* (Figure 5). The translation products of nine *FBX* genes (*At1g66310*, *At1g69630*, *At2g20380*, *At3g62230*, *At4g10400*, *At4g26340*, *At4g27050*, *At5g44980* and *At5g53840*) interacted with ASKs *2, 3* and/or *4* (Table S1), and showed correlation to the expression patterns of the *ASKs 3* and/or *4*, whose expression cannot be distinguished by microarray because of their high homology (93.3% identity in their open reading frames). Of the 9 *FBX* genes, seven (*At1g66310*, *At1g69630*, *At2g20380*, *At3g62230*, *At4g26340*, *At5g44980* and *At5g53840*), four (*At1g66310*, *At4g10400*, *At4g27050*, *At5g44980* and *At5g53840*), two (*At3g62230* and *At4g27050*), three (*At1g66310*, *At4g27050* and *At5g44980*) or one (*At4g27050*) *FBX* gene(s) showed a correlation in their expression to *ASKs 3* and/or *4* in pollen, endosperm and testa, inflorescence, callus and cultured cell, and root, respectively. Since At2g20380, At4g10400, At4g27050 and At5g53840 were observed to interact with only ASK3 by Y2H (Table S1), their expression profile implies a cooperative function between these FBX and ASK proteins in the tissues. Five *FBX* genes (*At1g60570*, *At2g22030*, *At3g04660*, *At4g02310* and *At4g29370*), whose translation products interacted with ASKs 13, 14 and/or 18 (Table S1), showed a correlative expression pattern to *ASK13* in endosperm and sperm cells. In addition, two *FBX* genes (*At2g22030* and *At3g04660*) had a correlation in their expression to *ASK13* in pollen. Since At2g22030, At4g02310 and At4g29370 were observed to interact only with ASK13 (Table S1), the expression profile implies a cooperative function between these FBX proteins and ASK13 in the tissues. With regard to *ASK14*, ten *FBX* genes (*At1g30790*, *At1g31080*, *At1g47730*, *At1g51290*, *At1g60570*, *At2g31470*, *At2g43270*, *At3g03360*, *At3g04660* and *At3g08750*), whose translation products interacted with ASKs 13, 14, 16 and/or 18 (Table S1), showed correlation to the expression pattern of *ASK14* in male tissues. Since At1g31080, At1g47730, At1g51290 and At2g31470 and At2g43270 were observed to interact only with ASK14 (Table S1), the expression profile implies that these FBX proteins and ASK14 co-act in the tissues. Two *FBX* genes, *At1g30790* and *At3g03360*, which interacted with ASK14 and ASK16 (Table S1), showed a similar expression pattern to *ASK16* as well as *ASK14*. *ASK16*, unlike *ASK14*, showed expression correlation to *At3g03360* in embryos and endosperm in addition to pollen, implying that At3g03360 is composed of two different types of SCF complexes by interacting with ASKs 14 or 16 and each complex works in distinct tissues. Tissues where the *At1g30790* gene correlated to the *ASK16* gene were similar to *ASK14* gene, implying that there is redundancy between the ASK14 and ASK16 proteins in their interactions with At1g30790. Three *FBX* genes (*At2g07140*, *At3g08450* and *At3g17490*), whose translation products interacted with ASKs 14 and/or 18 (Table S1), displayed similar expression patterns to *ASK18*. Of them, both *At3g08750* and *At3g17490* showed correlation to *ASK18* in male tissues. In addition, *At2g07140* showed correlative expression to *ASK18* in callus and cell culture. Since At2g07140 and At3g17490 were observed to interact only with ASK18, the expression profile implies a cooperative function between these FBX proteins and ASK18 in the tissues. *At3g22940*, whose translation product interacted with ASK18 and ASK19 (Table S1), had a correlative expression to *ASK19* in pollen and cell culture.

### Subcellular localization of FBX proteins

**Figure 6:**
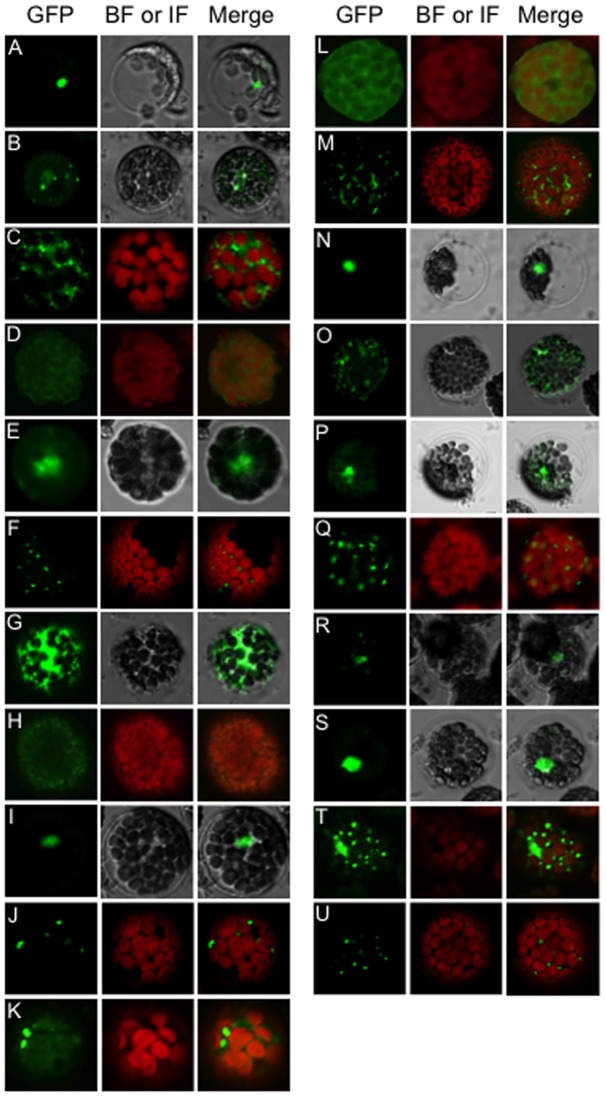
Subcellular localization of FBX proteins. GFP-fused FBX proteins; At1g21410 (A), At1g21760 (B), At1g23390 (C), At1g30790 (D), At1g47730 (E), At1g64840 (F), At1g67190 (G), At2g24250 (H), At2g25490 (I and J), At3g03360 (K), At3g04660 (L), At3g24760 (M), At4g02440 (N), At4g05460 (O and P), At4g27050 (Q and R), At5g21040 (S and T) and At5g52880 (U) were observed. BF, bright field; IF, intrinsic fluorescence.

For further understanding of FBX proteins, seventeen were randomly chosen, including four used in the BiFC analysis that were observed to interact with any ASKs (Table S1), to examine the subcellular localization of GFP-fused FBX proteins. As shown in Figure 6 and Table S1, GFP signals were observed in various intracellular compartments in protoplast cells. Two FBX proteins (At1g21410 and At4g02440) preferentially showed localization in the nucleus (Figure 6A and 6N). GFP signals of 3 FBX proteins (At1g30790, At1g67190 and At3g04660) were observed throughout the cytoplasm (Figure 6D, 6G and 6L), implying that they were localized in the cytosol or vacuole. Five proteins (At1g23390, At1g64840, At3g03360, At3g24760 and At5g52880) showed a preferential speckled localization in the cytoplasm (Figure 6C, 6F, 6K, 6M and 6U). Six (At1g21760, At1g47730, At2g25490, At4g05460, At4g27050 and At5g21040) localized in both the nucleus and the cytoplasm (Figure 6B, 6E, 6I–J and 6O-T), and speckled GFP signals were observed in five of them. The signal of FBX protein At2g24250 overlapped with intrinsic fluorescence (Figure 6H), implying localization in the chloroplasts.

## Discussion

FBX proteins have been shown by Y2H to have preferences for which ASK proteins they interact with. All the ASK proteins that interacted with large numbers of FBX proteins (ASKs 1–4 and 11–14) were classified as a cluster with ASK1 except ASK14 in a phylogenic tree in Figure 1B. The C-terminal region of human SKP1 interacts with a FBX protein SKP2 [23] and the ASK proteins tested have high homology to SKP1 at their C-terminal regions with the exceptions of ASKs 7 and 20 (data not shown). Nevertheless, a significant difference was shown in the interactive ability of ASK proteins with FBX proteins. This implies that other regions within ASK proteins may be responsible for the specificity against FBX proteins. The FBX domain is known to be the region that interacts with the SKP1 protein in human [23]. However, each ASK protein had a preference for FBX proteins that have been classified based on the distinct domains present in addition to the FBX or FBX-like domain. Since each distinct domain forms a specific three-dimensional structure, the domain may structurally affect in the specificity of FBX proteins for ASK proteins. In particular, FBX proteins carrying the Kelch domain had a remarkable specificity for ASK13 (Figure 2). It has been reported that repeated Kelch domains form a β-propeller tertiary structure [24]. Thus, its structure may support selective binding to ASK13, although to date there is no experimental evidence.

A large population of FBX proteins did not interact with any ASK protein, although they may interact with other ASK proteins which were not used in this study. It is known in many of other species that phosphorylation is necessary for interaction between FBX and ASK proteins [25]. FBX proteins that did not interact with any ASK protein may need certain modification *in vivo* such as phosphorylation or they may require additional proteins present in order to interact, although it is not excluded that the expression level of protein is not high enough to be detected by Y2H. Indeed, BiFC analysis showed FBX proteins interacted with more ASK proteins than did Y2H. These differences may support the necessity of modifications or other factors *in vivo* for some kinds of combination of FBX and ASK proteins to occur in plants. Alternatively, these FBX proteins may act differently to SCF complexes, although they have been categorized as FBX proteins.

From microarray data and *GUS* expression assays in previous reports [15], [21], it was revealed that each *ASK* gene had significant differences in the tissues where they were expressed and many of the *ASK* genes were particularly expressed in tissues related to male gametophyte and seed development. There are dramatic changes of intracellular environment in these tissues coupled to meiosis and mitosis, resulting in a rapid turnover of proteins. For these events to occur, many of the proteins may be regulated by Ub-mediated proteolysis through SCF complexes. Indeed, the amount of ubiquitinated proteins dramatically decreased during pollen maturation in maize [26]. In addition, several types of ubiquitinated proteins were reported in male tissues in plants [27]. Ub-specific proteases (UBPs) are one of the deubiquitinating enzymes, which release Ubs from ubiquitinated proteins in addition to primary translation products of Ubs. Doelling et al [28] revealed that UBP3/UBP4 was essential for pollen development, suggesting Ub-mediated protein regulation in pollen. Therefore, it is estimated that large amounts of SCF complexes could exist in these tissues. No striking difference was found in the numbers of expressed *FBX* genes in all tissues compared to the preferential expression of *ASK* genes in tissues related to male gametophyte and seed development, although there was a distinct expression pattern for each gene (Figure 4B–C). Thus, it is suggested that each FBX protein probably interacts with more ASK proteins in the tissues related to male gametophyte and seed development than in other tissues, making many types of SCF complexes.

Co-expression analysis in combination with Y2H and BiFC analysis will give more comprehensive understanding of FBX functions (Figures 3 and 5, and Table S1). The difference observed in between Y2H and BiFC analysis (Figure 3 and Table S2) may give dynamic behavior how SCF complexes were regulated not only by the combination of FBX and ASK proteins but also by their temporal amounts in each tissue and subcellular localization (Figures 5 and 6).

In this work, we have revealed that many FBX proteins interact with multiple ASK proteins. These results suggest that each FBX protein probably forms distinct multiple SCF complexes to handle large amounts of substrate proteins for ubiquitination. Moreover, comprehensive consideration of the interaction between FBX and ASK proteins, their localization and their gene expression could give helpful information for predicting the function of SCF complexes. Our results will be of use to the future investigation of Ub-mediated protein regulation through SCF complexes in plants.

## Materials and Methods

### Cloning of open reading frames (ORFs) of FBX and ASK protein families

Total RNAs were prepared using the NucleoSpin RNA plant kit (Macherey-Nagel) from Arabidopsis flowers, roots, siliques, stems, leaves and seedlings grown in either continuous white light or darkness for 1 week and then treated with RQ DNase I (Promega, Tokyo, Japan), following the manufacturer's instructions, to prevent contamination with genomic DNA. First strand cDNAs were synthesized from the total RNA as described previously [16]. Full-length cDNAs of the *FBX*s and *ASK*s were cloned by PCR from first strand cDNAs as described previously [16]. Each amplified PCR product was cloned into a Gateway pDONR207 vector by BP reaction (Gateway; Life Technologies Japan Ltd). Information on primers used in this experiment is given in Table S3. Plasmids carrying ORFs of *FBXs* (At1g68050, At1g78730, At2g25490, At2g42720, At3g16740, At3g18980, At3g23260, At3g57590, At4g38870, At5g39250, At5g43190, At5g49610 and At5g56370) and *ASKs* (1–4, 7–14 and 16–19) were constructed previously [15], [16].

### Y2H

Each *FBX* ORF fragment in pDONR207 was transferred to a pGBK-RC-Gateway vector [16] to fuse in-frame with the Gal4-DNA binding domain (Gal4-DB) by LR reaction (Gateway). Each *ASK* ORF fragment in pDONR207 was transferred to a pGAD-RC-Gateway vector [16] to fuse in-frame with the Gal4 activation domain (Gal4-AD) by LR reaction (Gateway). Plasmids carrying the ORFs of *FBXs* (At1g68050, At1g78730, At2g25490, At2g42720, At3g16740, At3g18980, At3g23260, At3g57590, At4g38870, At5g39250, At5g43190, At5g49610 and At5g56370) and *ASKs* (1–4, 7–14 and 16–19) were constructed previously [15], [16].

Transformation and mating of yeasts were performed as described previously [16]. Selection was performed on SD plates without leucine, tryptophan, histidine and adenine. Combinations of FBX and ASK that can grow on this selection plate were defined as ‘high strength of interaction’ (H). Selection was also performed on SD plates without leucine, tryptophan and histidine, and combinations of FBX and ASK that can grow on this selection plate was defined as ‘low strength of interaction’ (L). All combinations of 341 FBX and 19 ASK proteins were examined twice. When repeated twice the results were different, a third repetition was performed. After a colony formed, it was transferred onto a SD plate with *B*-galactoside to confirm the interaction.

### BiFC analysis

The ORFs of four *FBXs* (*At1g30790, At3g03360, At3g04660* and *At5g21040*) and 19 *ASKs* (*1*–*5, 7–8, 10–11*, *13*–*14,16*–*19, 20A* and *20B*) were amplified by PCR from plasmids inserted into pDONR207 as templates. Information on the primers used in this experiment is given in Table S3. Amplified *FBX* and *ASK* genes were inserted into a pSCYCE vector carrying the C-terminal half (174–328aa) of SCFP3A driven by the *CaMV 35S* promoter and a pSCYNE vector carrying the N-terminal half (1–173aa) of SCFP3A driven by the *CaMV 35S* promoter, respectively [29]. The generated plasmids were transformed into *Agrobacterium tumefaciens* strain GV3101, and equal volumes of the FBX and ASK *Agrobacterium* solutions were combined and infiltrated into 3-week-old leaves of *Nicotiana tabacum* as described previously [29]. After incubation at 28°C for 3 days, fluorescence of SCFP3A was observed by a fluorescent microscope (Olympus BX60 F5).

### Comparison of gene expression of *ASK* and *FBX* genes

GENEVESTIGATOR (https://www.genevestigator.com/gv/) was used to compare the gene expression of *ASKs* and *FBXs* in various tissues.

### Subcellular localization analysis of FBX proteins

Protoplasts were prepared from rosette leaves of 4-day-old Arabidopsis. Twenty leaves were cut into pieces 1 to 2 mm in length. These leaf pieces were treated in enzyme solution (1.5% cellulase R10, 0.4% macerozyme R10, 0.4 M mannitol, 20 mM KCl, 10 mM CaCl_2,_ 0.1% bovine serum albumin, 20 mM MES, pH 5.7) for 4 h at 25°C under dark conditions. The leaf pieces were filtrated through 50 *u*m nylon mesh and centrifuged at 800 rpm for 5 min. Pellets were gently resuspended in ice-cold solution A (0.4 M mannitol, 70 mM CaCl_2_, 5 mM MES, pH 5.7), and then incubated on ice for 30 min. The sample was then centrifuged at 800 rpm for 5 min, and the pellet was resuspended in 5 ml of MMg solution (0.4 M mannitol, 15 mM MgCl_2_, 4 mM MES, pH 5.7) producing a protoplast suspension.

Each ORF fragment of the FBXs in pDONR207 was transferred by LR reaction (Gateway) to pBE2113-GW [30] carrying GFP and a 35S CaMV promoter. Each PCR fragment containing the promoter, FBX and GFP was amplified from the FBX::GFP plasmids using primers (pBig-F and pBig-R; Table S). Information on the primers used in this experiment is given in Table S3. For transformation the PCR fragments were incubated with 100*u*l of protoplast solution and 110*u*l of polyethylene glycol (PEG) solution (50% PEG4000, 0.25 M mannitol, 0.125 M Ca(NO_3_)_2_) at room temperature for 30 min. The sample was gently mixed with 10 ml of solution A and centrifuged at 800 rpm for 5 min. The pellet was resuspended in 4 ml of Murashige and Skoog (MS) solution (1 x MS salt containing B5 vitamins, 0.4 M mannitol, 4 mM MES, pH 5.7). After incubation at 25°C for 10 h in darkness, GFP fluorescence was observed by a confocal microscope (Zeiss Laser Scanning Microscope LSM700).

## Supporting Information

Figure S1 **Comparison of expression patterns of** ***ASK*** **genes in various tissues by GENEVESTIGATOR.** ATH1: 22k array was used as the platform. Microarray was performed in tissues of callus (1), cell culture/primary cell (2), sperm cell (3), protoplast (4), guard cell protoplast (5), mesophyll cell protoplast (6), root protoplast (7), root cap protoplast (8), columella protoplast (9), lateral root cap protoplast (10), root epidermis and lateral root cap protoplast (11), root cortex protoplast (12), root endodermis and quiescent center protoplast (13), root stele protoplast (14), root phloem protoplast (15), root xylem protoplast (16), root cortex, endodermis and quiescent center protoplast (17), root epidermis protoplast (18), root epidermal atrichoblast protoplast (19), root culture (20), seedling (21), cotyledon (22), hypocotyl (23), radicle (24), imbibed seed (25), shoot apical meristem (26), inflorescence (27), flower (28), pistil (29), carpel (39), ovary (31), ovule (32), stigma (33), petal (34), sepal (35), stamen (36), anther (37), pollen (38), abscission zone (39), pedical (40), silique (41), replum (42), seed (43), embryo (44), endosperm (45), micropylar endosperm (46), peripheral endosperm (47), chalazal endosperm (48), testa (49), general seed coat (50), chalazal seed coat (51), suspensor (52), stem (53), developing meristemoid zone (54), node (55), shoot apex (56), cauline leaf (57), rosette (58), juvenile leaf (59), adult leaf (60), petiole (61), senescent leaf (62), hypocotyl (63), xylem (64), cork (65), leaf primordial (66), stem (67), axillary bud (68), axillary shoot (69), shoot apex (70), roots (71), primary root (72), root tip (73), meristematic zone (74), elongation zone (75), root hair zone (76), stele (77), pericycle (78) or lateral root (79). Percentage expression potential is shown from 0 (white) to 100 (dark brown) % according to the six-grade system.
(XLSX)

Figure S2 **Comparison of expression patterns of** ***FBX*** **genes in various tissues by GENEVESTIGATOR.** ATH1: 22k array was used as the platform. Microarray was performed in tissues of callus (1), cell culture/primary cell (2), sperm cell (3), protoplast (4), guard cell protoplast (5), mesophyll cell protoplast (6), root protoplast (7), root cap protoplast (8), columella protoplast (9), lateral root cap protoplast (10), root epidermis and lateral root cap protoplast (11), root cortex protoplast (12), root endodermis and quiescent center protoplast (13), root stele protoplast (14), root phloem protoplast (15), root xylem protoplast (16), root cortex, endodermis and quiescent center protoplast (17), root epidermis protoplast (18), root epidermal atrichoblast protoplast (19), root culture (20), seedling (21), cotyledon (22), hypocotyl (23), radicle (24), imbibed seed (25), shoot apical meristem (26), inflorescence (27), flower (28), pistil (29), carpel (39), ovary (31), ovule (32), stigma (33), petal (34), sepal (35), stamen (36), anther (37), pollen (38), abscission zone (39), pedical (40), silique (41), replum (42), seed (43), embryo (44), endosperm (45), micropylar endosperm (46), peripheral endosperm (47), chalazal endosperm (48), testa (49), general seed coat (50), chalazal seed coat (51), suspensor (52), stem (53), developing meristemoid zone (54), node (55), shoot apex (56), cauline leaf (57), rosette (58), juvenile leaf (59), adult leaf (60), petiole (61), senescent leaf (62), hypocotyl (63), xylem (64), cork (65), leaf primordial (66), stem (67), axillary bud (68), axillary shoot (69), shoot apex (70), roots (71), primary root (72), root tip (73), meristematic zone (74), elongation zone (75), root hair zone (76), stele (77), pericycle (78) or lateral root (79). Percentage expression potential is shown from 0 (white) to 100 (dark brown) % according to the six-grade system.
(XLSX)

Figure S3 **Comparison of expression patterns of** ***FBX*** **genes in various tissues by GENEVESTIGATOR.** AGRO1: AGRONOMICS whole genome tiling array was used as the platform. Microarray was performed in tissues of seedling (1), inflorescence (2), raceme (3), flower (4), shoot (5), rosette (6), leaf (7), juvenile leaf (8) or adult leaf (9). Percentage expression potential is shown from 0 (white) to 100 (dark brown) % according to the six-grade system.
(XLSX)

Table S1 **Information of cloned FBXs and summaries of Y2H, BiFC experiments, microarray comparison and subcellular localization.**
(XLSX)

Table S2 **Comparison between** ***in vitro*** **and** ***in vivo*** **interaction of ASKs and 3 FBXs chosen from** **Figure 5** **.**
(XLSX)

Table S3 **Primers used in this study.**
(XLSX)
